# Adult cell leukemia (ATL) in a patient with HTLV infection - case presentation

**DOI:** 10.1186/1471-2334-14-S4-P50

**Published:** 2014-05-29

**Authors:** Gabriel Colțan, Cristina Marin, Mariana Anghel, Tatiana Colțan, Mihai Olariu, Ana Maria Vlădăreanu, Elena Ambrus, Irina Pârvu

**Affiliations:** 1 National Institute for Infectious Diseases “Prof. Dr. Matei Balş”, Bucharest, Romania; 2 University Emergency Hospital, Bucharest, Romania

## 

Infections with human retroviruses, others than the HIV virus, are not very well known in Romania, despite the fact that screenings for HTLV I and II are constantly performed in blood donation centers. The authors draw attention on the possible severe consequences that may occur in chronic infections with these viruses by presenting the case of a 35 year-old male, who was known to be infected with HTLV I virus for several years and who consequently developed adult cell leukemia.

The authors also plead for the development of an algorithm for the detection, surveillance, monitoring and treatment of HTLV infections, throughout interdisciplinary cooperation between infectious disease specialists, hematologists, dermatologists.

The authors strongly recommend the development of means to promote prevention by: securing blood transfusions; forbidding breastfeeding in women who tested positive for HTLV; using condoms in order to prevent sexual transmission in discordant couples.

